# Reviews

**Published:** 1882-02

**Authors:** 


### Reviews.

**Landmarks, Medical and Surgical.** By LUTHER HOLDEN, Consulting Surgeon to Saint Bartholomew's and the Foundling Hospitals, etc. Assisted by James Shuter, M. A. Camb, Assistant Surgeon to the Royal Free Hospital, etc. From the third English edition, with additions by William W. Keen, M. D., Professor of Artistic Anatomy in the Pennsylvania Academy of the Fine Arts, etc. Philadelphia: Henry C. Lea's Son & Co. 1881.

It is as important for the student of anatomy to make himself acquainted with the exterior form of the living human body as to dissect the cadaver. Hand and eye must work together if we want to insure ourselves against a faulty diagnosis. How often we see that a dislocation is overlooked because the surgeon is not familiar with the outer appearance of the human body This little book is a good guide to the study of this branch of anatomy, and will be found of value not only by the student, but also by the practitioner.

---

**Suppression of Urine**; Clinical Descriptions and Analysis of Symptoms. By E. P. FOWLER, M. D. Ninety-three clinical cases, with illustrations, tables, and diagrams New York: William Wood & Co. 1881.

This book is an entertaining monography, originally presented as a paper to the New York Medico-Chirurgical Society. The author reports first a very interesting case of anuria of ten days' duration, produced by pressure of a cystic degeneration of left kidney on the right renal artery, and gives as his impression "that sudden and total urinary suppression, in absence of other acute illness or of poisoning, is nearly always co-existent with the presence—physiologically at least—of but one kidney." The author has collected 93 cases from the literature of the last 100 years, and arranged them in tables according to the causes given statistics respecting symptoms, duration, age, sex, etc. Altogether the little book is a very valuable contribution to the medical libraries, so much more, as it, as far as we know, is the only one on this subject.

**The Opium Habit and Alcoholism.** A treatise on the habits of opium and its compounds: alcohol, chloral hydrat, chloroform, bromide of potassium and canabis indica, including their therapeutical indications, with suggestions for treating various painful complications. By DR. FRED. HEMAN HUBBARD. New York: A. S. Barnes & Co.

This remarkable title is borne by a neatly bound book of 259 pages, which the author claims in his preface was written for the benefit of the profession. It is in fact a sensational description of the evils of the opium habit, mixed up with all sorts of topics foreign to the subject, and written in a style to please the vulgar rather than the professional reader. The book is valueless to the medical man, injurious in its influences upon the layman, and profitable only (if at all) to the author as an advertisement, for which it is probably intended.

---

**Aids to Rational Therapeutics.** By T. MILNER FOTHERGILL, M. D., M. R. C. P. New York: G. P. Putnam's Sons. Paper, 25 cts.; cloth, 50 cts.

This little book is one of the student's aids series, and is one of the best of them. The young graduate particularly will find many hints in this little book which will help him greatly when first attending patients himself.

---

**A Pocket Book of Physical Diagnosis for the Student and Physician.** By DR. EDWARD T. BRUEN, one of the Physicians to the Philadelphia Hospital, &c., &c., with wood engravings. Philadelphia: Presley Blakiston. Price, $2.00.

This volume aims to give to the student and physician a condensed reliable manual on physical diagnosis. The arrangement is original; the illustrations are drawn especially for the work, and the author has succeeded in producing a book of considerable merit. It is a work which we would especially recommend to students, as of value in welding together information gleaned from didactic lectures and reading with that obtained by observation at the bedside.

**The Physician's Clinical Record**, for hospital and private practice, with memoranda for examining patient's temperature, chests, etc. Philadelphia: D. G. Brinton.

Accurate records of his more important cases are of value not only to the physician himself, but to the profession; but it is practically impossible for him to keep such records with the needed accuracy and desirable brevity, unless he uses a regular form and one that he can have always with him. To this end the present volume contributes. It is small enough to be carried in the pocket, complete enough for all the essential entries and comments, and large enough for over a hundred cases. A small stencil plate accompanies it to be used for sketching an outline of the thorax and abdomen, in which may be marked by shading the seats of effusion, the size of organs, the position of tumors, etc.

---

**Chemical Analysis of the Urine**, based in part on "Casselmann's Analyse des Harnes." By E. F. SMITH, Ph. D., Professor of Chemistry in Muhlenberg College, and John Marshal, M. D., Demonstrator of Chemistry, University of Pennsylvania. Illustrated Philadelphia: Presley Blakiston. Price, $1 00.

There is no want of hand-books on urinalysis. Those who are not already supplied will find this one of the best of the smaller works; the arrangement is good and the illustrations excellent.

---

**Lectures on Electricity** (Dynamic and Franklinic) in its relations to medicine and surgery. By A. D. ROCKWELL, M D., Electro-Therapeutist to the New York State Woman's Hospital, &c. New York: Wm. Wood & Co.

This is a second edition of a manual which has met with general favor. A number of additions have been made; especially a description of the galvanic accumulator for storing electricity for surgical uses, and of the induction-balance for locating the position of bullets in the body; also a lecture on Franklinic Electricity. Those who have not the larger work of Beard & Rockwell will find this almost a necessity if they use electricity at all.

